# From safety to agency: experiences of self-admission among patients with diverse mental health needs

**DOI:** 10.1080/17482631.2026.2641161

**Published:** 2026-03-11

**Authors:** Emelie Allenius, Mattias Strand, Joachim Eckerström, Alexander Rozental, Pernilla Omerov, Sigrid Salomonsson

**Affiliations:** a Centre for Psychiatry Research, Department of Clinical Neuroscience, Karolinska Institutet & Stockholm Health Care Services, Region Stockholm, Stockholm, Sweden; b Division of Nursing, Department of Neurobiology, Care Sciences and Society, Karolinska Institutet, Stockholm, Sweden; c Department of Health, Education and Technology, Luleå University of Technology, Luleå, Sweden

**Keywords:** Person-centred care, self-admission, mental health conditions, psychiatric inpatient care, psychiatric nursing, thematic analysis

## Abstract

**Introduction:**

Self-admission models allow patients to directly contact their psychiatric ward for brief inpatient care based on self-assessed need. To ensure equitable access across all psychiatric services, a broadly applicable, transdiagnostic model for self-admission was developed in Region Stockholm - Sweden's largest public healthcare provider. This study aimed to explore patients' experiences of access to self-admission and its impact on everyday life during mental health problems.

**Methods:**

Sixteen semi-structured interviews were analyzed using thematic analysis.

**Results:**

The analysis yielded one overarching theme, *From safety to agency*, and three themes: *Sense of security*, *Care that supports* and *Facilitating recovery.* The findings illustrate that the self-admission model fostered safety and autonomy and was perceived as facilitating coping strategies, crisis plans, and greater self-awareness and self-management, helping prevent deterioration and reducing emergency care needs. The model also supported maintaining meaningful routines and social connections. Although generally perceived as empowering, some participants struggled with increased autonomy and emphasized the need for greater involvement of relatives.

**Discussion:**

The self-admission model appears to effectively promote person-centred care and personal recovery. The study supports previous research as well as demonstrates that a transdiagnostic self-admission model can assist patients with mental health conditions.

## Introduction

Patients experiencing mental health problems often face complex and long-term challenges that affect everyday functioning and quality of life, underscoring the need for person-centred mental health care (Gabrielsson et al., 2020; Socialstyrelsen, 2025). Key components of such care are the redistribution of decision-making power toward the patient, emphasizing mutual respect, trust, shared decision-making, and responsiveness to the patient’s needs, preferences, and capacities (Ahlstrand et al., 2024; McCance & McCormack, 2025). Person-centered care has been linked to improved service access, better care organization, higher quality, more effective healthcare (Hormazabal-Salgado et al., 2024; Khosravi et al., 2024), and increased patient satisfaction (Ahlstrand et al., 2024; McCance & McCormack, 2025; Staniszewska et al., 2019). In Sweden, person-centred care is an explicit goal in legislation and national policy (Hälso- och sjukvårdslag [HSL], 2017; Socialstyrelsen, 2025). Despite this, implementation within mental health services has been challenging. Barriers described include limited time and resources, uncertainty regarding roles and responsibilities, entrenched clinical routines, and difficulties translating person-centred principles into everyday clinical practice. This highlights a gap between policy ambitions and clinical reality, emphasizing the need for interventions that operationalize person-centred care in standard psychiatric settings (Ahlstrand et al., 2024; Forsgren et al., 2025).

In standard psychiatric care pathways in Sweden, inpatient admission typically follows a clinician-led assessment, usually conducted in psychiatric emergency departments but also in outpatient services. While patients may seek help during crisis, admission decisions rest with healthcare staff (Hälso- och sjukvårdslag [HSL]; Socialstyrelsen, 2026). Previous research has shown that such encounters are frequently experienced by patients as demanding and, at times, invalidating, particularly during acute phases of mental illness. Patients have reported feeling scrutinized, not taken seriously, and having limited influence over decisions affecting their care, which may contribute to delayed help-seeking and symptom escalation (Harris et al., 2016; Molin et al., 2016; Silva et al., 2023), and ultimately result in a perceived lack of safety (Vogt et al., 2024). Negative experiences of psychiatric care may persist beyond hospitalization and adversely affect everyday life (Silva et al., 2023). These challenges highlight the need for person-centred care approaches that not only address symptoms but also strengthen patient’s sense of agency and autonomy, support decision-making and enable active participation in everyday life (Bergamin et al., 2022; Khosravi et al., 2024). Self-admission has been proposed as one way to operationalize essential principles of person-centred care - such as autonomy, shared responsibility, and timely access to care tailored to individual needs and preferences - across the continuum of psychiatric services (Eckerström et al., 2020; Socialstyrelsen, 2021).

### Self-admission models

Several self-admission models have been developed and adopted in mental health services across Scandinavia, the Netherlands, and Australia, by various names, including *brief admission* (Helleman et al., 2014a), *patient-controlled admission* (Sigrunarson et al., 2017), *patient-controlled hospital admission* (Thomsen et al., 2018), *open borders program* (Mortimer-Jones et al., 2016), and *patient-initiated brief admission* (Värnå et al., 2025). The aim of these models is to enhance patient involvement, autonomy, and access to care by providing timely preventive support during periods of mental health crisis and symptom exacerbation. All self-admission models are add-on interventions to standard care, and common components include patients’ direct access to brief inpatient care, thereby bypassing the emergency department. Moreover, it includes self-assessment of care needs, a pre-agreed care arrangement, and the promotion of autonomy and self-efficacy. When a patient has an agreement in place and opts for admission, staff are not permitted to challenge the decision (Helleman et al., 2014a; Mortimer-Jones et al., 2016; Sigrunarson et al., 2017; Thomsen et al., 2018; Värnå et al., 2025). Studies on the effects of these models indicate a reduction in both overall inpatient care use and compulsory admissions (Daukantaite et al., 2025; Hagsäter et al., 2025; Nyttingnes et al., 2021; Sigrunarson et al., 2017; Skott et al., 2021; Strand et al., 2020; Westling et al., 2019), as well as a reduction in the length of individual hospital stays, which may be linked to a shift from longer inpatient care utilization to increased outpatient care utilization (Eckerström et al., 2024). Staff describe more equal and trust-based care relationships when patients have access to self-admission, and express satisfaction with the model for enhancing support of patient self-management (Eckerström et al., 2019; Mortimer-Jones et al., 2019). According to qualitative studies of patients lived experiences, access to these models appears to enhance patients' autonomy, sense of security, prevent the negative spiral of worsening symptoms and provides a solution that helps patients maintain a sense of control. Furthermore, patients have described feeling more welcomed and respected during admissions and encouraged by staff to actively participate in their care (Värnå et al., 2025). At the same time, studies on patients’ experiences have identified challenges related to the use and implementation of self-admission models, including uncertainty during early phases of access, difficulties in changing help-seeking behaviors, and experiences of insufficient staff support or unclear boundaries (Värnå etal., 2025). These findings highlight the importance of further exploring how such models are experienced in practice and how they may be refined to better support patients’ everyday lives.

### Self-admission in Stockholm, Sweden

The self-admission models share core components but differ in structure, content, and target population (Helleman et al., 2014a; Mortimer-Jones et al., 2019; Paaske et al., 2021; Thomsen et al., 2018). An evaluation from the Swedish National Board of Health and Welfare (Socialstyrelsen) showed that self-admission models had been implemented in 18 of Sweden’s 21 regions, although, there was considerable regional variation in both its design and the patient groups served (Socialstyrelsen, 2021). In 2015, three different self-admission models were introduced in Region Stockholm, Sweden's largest public healthcare provider serving approximately 2.5 million residents (Region Stockholm, 2025). These small-scaled project targeted patients diagnosed with psychosis, anorexia nervosa, and emotional instability and/or self-harm with great healthcare needs. Based on the overall positive effects, a political decision was made in 2019 by the healthcare authorities in Region Stockholm to implement a generic model for self-admission across all mental health services, replacing the initial projects (Smitmanis Lyle et al., 2022). The term “generic” refers to the model’s transdiagnostic structure, enabling use across psychiatric services while still allowing individualized admission agreements. The generic self-admission model was developed to promote consistency and equity across services. The implementation includes child- and adolescents, as well as adult psychiatry (with the exception of substance use services) regardless of diagnosis. Further, it involves both government-run healthcare and private providers operating within publicly funded healthcare. In addition to implementing the generic self-admission model, the health care services decided to evaluate the model from the user perspective as well as by measuring its effects (Smitmanis Lyle et al., 2022).

Overall, previous research has highlighted predominantly positive patient experiences with access to self-admission models (Värnå et al., 2025). While various self-admission models are currently in use, no previous qualitative studies have, to our knowledge, explored patients’ experiences of a transdiagnostic, region-wide self-admission model implemented across services and diagnostic groups. To address this gap, the present study aimed to explore adult patients’ experiences of having an agreement within the generic self-admission model and its impact on their everyday lives. Accordingly, the study investigated patients’ experiences of access to and the opportunity to use self-admission, regardless of actual use.

## Method

### Design

To explore patients experiences of having an agreement within the generic self-admission model and the subsequent impact on their everyday living, an inductive descriptive design using thematic analysis was deemed appropriate, as it allowed a flexible, data-driven exploration of the phenomenon (Braun & Clarke, 2006). The Consolidated Criteria for Qualitative Research (COREQ) guided the proceedings (Tong et al., 2007). This study is part of the broader ongoing evaluation of the self-admission model outlined above.

### Intervention—the generic self-admission model

**Figure 1.**
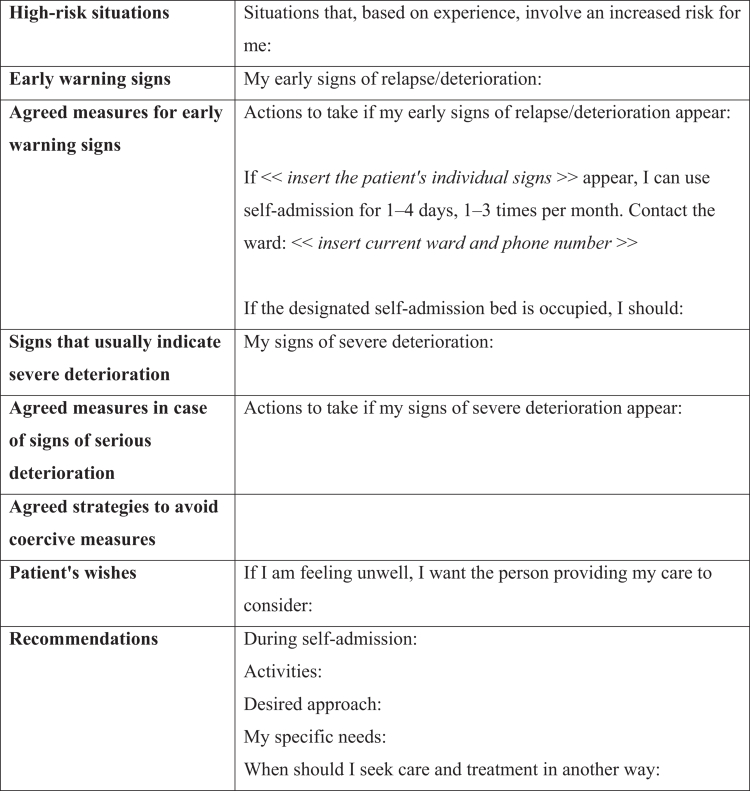
Agreement within the generic self-admission model, added to the patient’s crisis plan in the medical record.

The study aims to explore adult patients' experiences of having an agreement within the generic self-admission model, which is offered to patients regardless of psychiatric diagnosis on the condition that they meet the following criteria: maintaining an ongoing outpatient contact; an established individual care plan and crisis plan; inpatient care utilization in the past 12 months; and a continued need for inpatient care. Additionally, patients must express a desire for access to self-admission and demonstrate an understanding of its purpose. Patients granted an agreement maintain ongoing contact with outpatient services and are therefore referred to as "patients" in this study. The standard agreement, developed collaboratively between the patient and healthcare providers, allows patients to self-refer to a designated inpatient ward for up to four days at a time, up to three times per month. However, the agreement allows for individual adaptations based on the patient’s specific nursing needs and wishes. Admission and discharge are performed by registered nurses who have a formal temporary delegation to perform these duties. The agreement is added to the crisis plan in the medical record (as shown in Figure 1) and remains valid for 12 months, after which a joint evaluation involving the patient and staff from both outpatient and inpatient services determines whether it should be renewed and, if so, whether any modifications are needed. The intervention requires collaboration between inpatient and outpatient care services, and each unit implementing the model has designated self-admission staff responsible for its delivery (Smitmanis Lyle et al., 2022).

### Setting

Region Stockholm provides 40 psychiatric inpatient beds per 100,000 inhabitants. In 2023, 8,5% of adults utilized psychiatric services (Sveriges kommuner och regioner [SKR], 2023). The generic self-admission model is currently being implemented across all psychiatric clinics. Within adult psychiatry, this includes approximately ten departments, which collectively encompass 40 inpatient wards and 70 affiliated outpatient clinics. To date, 25 wards and 60 outpatient clinics have adopted the model, and approximately 500 patients have access to the model. These units specialize in caring for a range of psychiatric conditions, including schizophrenia and other psychotic disorders, affective disorders, personality disorders, and eating disorders.

### Participants and recruitment

The inclusion criterion for participating in the study was at least six months’ access to the generic self-admission model within adult psychiatry. No exclusion criteria were applied. The aim was to recruit a diverse sample of participants in terms of mental health conditions, age, gender, and experience with self-admission. Participants were initially recruited from four purposively selected outpatient clinics in different areas and services in Stockholm, to obtain a heterogeneous sample of adults with diverse mental health conditions (Braun & Clarke, 2013). Initial study information was emailed to department heads and designated self-admission staff at each outpatient unit and all agreed to participate. Primary contacts in the outpatient clinics were asked to provide both verbal and written information to potential participants. Patients who expressed interest in participating were referred to EA and JE, who subsequently provided additional information. Having primary contacts invite patients ensured that interviews were not conducted during acute symptom deterioration or ongoing inpatient care. Out of 21 eligible patients, seven expressed interest and were contacted to schedule interviews. To increase both the number and diversity of participants a convenience sampling was applied by distributing written study information to all designated self-admission staff at each outpatient unit (Braun & Clarke, 2013), with a request that they inform patients who met the inclusion criteria. As a result, ten additional individuals expressed interest and were scheduled for interviews. Data collection was concluded at 17 interviews.

One participant later withdrew from the study, expressing concerns about their responses and discomfort with the prospect of others reading them. Consequently, 16 interviews were included in the analysis. Of these participants, 10 were women and 6 were men, ranging in age from 21 to 63 years, with a mean age of 45. Among the participants, 12 had used their agreements to admit to inpatient care. Table I provides an overview of the demographic, clinical, and interview-related characteristics of the participants.

**Table I.** Demographic, clinical, and interview-related characteristics of the participants.

Gender	Age	Self-reported diagnostic group (*and diagnosis*)	Self-admission usage (yes/no)	Interview length (min:sec)
Female	47	Affective and anxiety disorders (*Recurrent depressive disorder, panic disorder, anxiety, social anxiety disorder*)	Yes	44:29
Male	62	Stress-related disorder (Exhaustion disorder*)	No	38:22
Female	40	Affective, eating, and personality disorders (Exhaustion disorder*, Recurrent depressive disorder, Borderline personality disorder, Anorexia Nervosa)	Yes	51:43
Female	34	Affective and neurodevelopmental disorders *(Recurrent depressive disorder, Borderline personality disorder, Attention-Deficit Hyperactivity Disorder)*	Yes	35:24
Male	55	Bipolar and anxiety disorders *(Bipolar Disorder (Unspecified), Depressive disorder, Generalized Anxiety Disorder)*	No	43:14
Male	54	Affective and anxiety disorders (*Depressive disorder, Panic Disorder)*	Yes	58:14**
Female	21	Personality disorder *(Borderline personality disorder)*	No	53:19
Male	39	Psychotic disorder *(Paranoid Schizophrenia)*	Yes	35:24**
Male	32	Psychotic disorder *(Schizoaffective Disorder)*	Yes	26:36**
Female	50	Bipolar and neurodevelopmental disorders *(Attention-Deficit Hyperactivity Disorder, Bipolar Disorder (Unspecified))*	Yes	42:18**
Female	37	Neurodevelopmental, trauma-related, anxiety and personality disorders *(Attention-Deficit Hyperactivity Disorder, Generalized Anxiety Disorder, Borderline personality disorder, Post-Traumatic Stress Disorder)*	Yes	52:28**
Male	51	Neurodevelopmental, and affective disorder *(Autism Spectrum Disorder, Depressive disorder)*	Yes	33:03**
Female	54	Neurodevelopmental, trauma-related, anxiety and personality disorders *(Attention-Deficit Hyperactivity Disorder, Generalized Anxiety Disorder, Borderline personality disorder, Post-Traumatic Stress Disorder)*	Yes	19:22
Female	40	Bipolar, anxiety and stress-related disorders (Exhaustion disorder^*^, Generalized Anxiety Disorder, Bipolar II Disorder)	Yes	46:43**
Female	36	Neurodevelopmental, personality and stress-related disorder (Attention-Deficit Hyperactivity Disorder, Borderline personality disorder, Exhaustion disorder*)	No	91:28
Female	63	Affective, neurodevelopmental and eating disorder *(Anorexia Nervosa, Autism Spectrum Disorder)*	Yes	31:47**

^*^ A diagnosis previously used in the Swedish healthcare system but not internationally recognized.

^**^ Interviews conducted by telephone.

### Data collection

Data was collected through semi-structured interviews guided by an interview guide (Appendix 1), designed to explore patients’ experiences of the generic self-admission model in Region Stockholm. The interview guide was informed by key concepts of person-centred care to explore while maintaining openness and flexibility (McCance & McCormack, 2025). It was also reviewed by a representative of the Swedish Partnership for Mental Health (Nationell Samverkan för Psykisk Hälsa, NSPH), a national network of patient, user, and family carer organizations working to strengthen involvement and improve the quality of mental health services (NSPH, 2026). The interviews began with open-ended questions encouraging participants to reflect on their life situations, providing insights into their personal experiences, hopes, aspirations, and everyday challenges. Subsequently, open-ended questions were posed regarding their experiences with self-admission, such as “What are your experiences with the generic self-admission model?”. Follow-up prompts, including “Please tell me more,” were used when appropriate to encourage elaboration. More focused questions were introduced when participants had difficulty reflecting or elaborating on their responses (Price, 2002). The semi-structured interviews were conducted by EA (female, RPN PhD student), JE (male, RPN PhD), and two MSc students in psychology (males), who were involved in data collection as part of their master’s thesis. All interviewers had prior experience working in specialized mental healthcare. Interviews were scheduled directly with the participants, who were offered face-to-face interviews whenever possible, or telephone interviews if they preferred or could not attend in person. Eight interviews were conducted face-to-face, either at the participants’ outpatient unit or at the research team’s office, and the remaining eight were conducted by telephone. Only the interviewer and participant were present during each interview. Interviews were conducted between December 2022 and April 2024, with durations ranging from 19 to 91 minutes and a mean duration of 43:59 minutes. Field notes were taken at the end of each interview to summarize key impressions and reflections. All interviews were audio-recorded and transcribed verbatim by EA and the MSc students.

### Analysis

The interviews were analyzed inductively, using Braun and Clarke’s thematic analysis (Braun & Clarke, 2006). All authors had experience in qualitative methodology. The primary analysis was conducted by EA in collaboration with PO. Initially, the transcriptions were read individually multiple times to gain a comprehensive understanding of the material. During and after familiarization, initial ideas for codes and themes were noted and subsequently discussed between EA and PO to ensure a shared early interpretation of the material. EA and PO then independently started generating initial codes at a semantic level, guided by the study’s objective. The coded data extracts ranged from a few words to a few sentences, with additional surrounding text included when needed to ensure sufficient contextual understanding. The coding was conducted using NVivo software. The dataset was coded in Swedish, and the initial codes were iteratively refined to accurately reflect the underlying data. Similar codes were then grouped into potential themes. This stage was initially conducted independently, where similar codes were first inductively clustered, then repeatedly moved, merged, and refined into larger theme-aligned groups. Once a preliminary set of subthemes and themes had been identified, EA and PO engaged in joint discussions with MS and JE, who had read the interview transcripts and provided feedback based on their interpretations of the material. The coded data and transcripts were re-read to ensure that the identified themes accurately reflected the interviews. Following translation of the preliminary findings into English and circulation to the co-authors, joint discussions refined the terminology and phrasing to consensus, and their feedback further informed revisions to themes and subthemes, including renaming as well as selective relocation or merging of specific subthemes.

After 13 interviews had been conducted and transcribed, the preliminary notes and ideas indicated that the dataset already offered substantial richness in relation to the study aim. Four additional interviews were nevertheless carried out, which added further nuance and depth to the developing insights, although they did not shift or expand the central patterns of meaning. Data collection was therefore completed at 17 interviews, based on a joint decision by EA, PO, and SS. Although one participant later withdrew, leaving 16 interviews for analysis, the dataset was considered rich and sufficient to address the research question meaningfully (Braun & Clarke, 2019). Selected quotations were used to illustrate and support the themes. Square brackets indicate references to participants or clarifications added by EA and PO.

Reflexivity was integrated throughout the thematic analysis to enhance methodological rigor. The research team engaged in continuous critical reflection throughout the research process. Regular team meetings were held to examine how pre-understandings might influence analytic decisions and data interpretation during both data collection and analysis. The authors represented diverse professional backgrounds and had clinical experience working with patients with mental health conditions across various care settings. Their experience with self-admission ranged from no prior clinical exposure to extensive practice. To ensure transparency, the researchers maintained a reflexive journal documenting analytic decisions, reflections on the researcher’s role, the interview context, personal preconceptions, and emerging analytic insights. To support dependability and confirmability, all raw data—including audio-recorded interviews, verbatim transcripts, methodological notes, and reflexive memos—were accessible to all members of the research team throughout the analytic process.

### Ethical considerations

The study was approved by the Swedish Ethics Review Authority (Dnr: 2020-06498), which is the government agency responsible for reviewing and granting ethical approval for research in Sweden. The study was conducted in accordance with the principles outlined in the Declaration of Helsinki. All participants received both oral and written information about the study, including its purpose, the voluntary nature of participation, assurance that participation would not affect their care, and their right to withdraw at any time without consequence. Additional details were provided regarding how the results would be presented, and participants were given the opportunity to ask questions. Contact details for support were provided in case they required further information or emotional support during the research process. Those who agreed to participate signed a written informed consent form. Confidentiality was ensured by anonymizing all transcripts and securely storing data. There were no prior personal or professional relationships between the interviewers and participants, and the researchers had no personal interests in the study.

## Results

Participants’ experiences of the generic self-admission model, and its impact on their daily lives were grouped into one overarching theme; *From safety to agency*, three themes; *Sense of security*, *Care that supports* and *Facilitating recovery.* Each theme includes two or three sub-themes, as presented in Table II.

**Table II.** Table of overarching theme, themes, and sub-themes.

*Overarching theme*	From safety to agency		
*Theme*	**1. Sense of security**	**2. Care that supports**	**3. Facilitating recovery**
*Sub-theme*	*1.1 Reliable and constructive support*	*2.1 Bridging care and everyday life*	*3.1 Strengthening and challenging autonomy*
	*1.2 Avoiding care-related suffering*	*2.2 Supportive inpatient care*	*3.2 Promoting self-awareness and help-seeking*
	*1.3 Relief for relatives*		*3.3 Empowerment and confidence in own resources*

### From safety to agency

Participants' experiences of self-admission can be understood as a process from safety to agency. Initially, the model offered a sense of safety and predictability. Over time, participants described how this safety enabled greater autonomy, the development of coping strategies, and increased confidence in managing everyday life. This progression is captured in three interconnected themes below.

*1. Sense of security*

Most participants reported an increased sense of security after enrolling in the self-admission program. The elements identified were grouped into the following subthemes.

*1.1 Reliable and constructive support*

Knowing help was just a phone call away provided a sense of security for the participants, and this was reported by both patients who had used self-admission and those who had not. Participants described access to self-admission as "*a safety button*", a "*lifeline to use if it gets really bad*", and "*a tool in my toolbox, something I can turn to when my emotions become too overwhelming*". The option to self-admit to a designated inpatient ward with familiar staff, routines and environment added to the security.

*“when I do ask for help, it’s really serious. And the people on the ward, for example, know that. When I’m in such a bad place, they... [in the ED]... think that I’m not sick enough to be admitted because I’m so calm and composed and speak clearly on the outside, but it’s burning inside of me” (7)*

Participants described calling the designated ward and receiving phone support to help de-escalate difficult situations and emotions. In some cases, phone support alone was sufficient, making admission unnecessary. Self-admission was initiated when challenges became too overwhelming. Knowing that supportive care was available helped reduce emotional distress. The enhanced security was described as sustaining and strengthened the confidence to tolerate moments of discomfort, resulting in greater resilience. Some participants reported needing less inpatient care since access to self-admission, linking this improvement to the model alone or together with treatments like dialectical behavior therapy.

*“… I can if I need to, and that has probably made me… not need it as much, if you know what I mean… because I feel like, ah well, I’ll see how things are tomorrow, today is a bit tough but… and then it has ended up like, yeah… no, I don’t need to admit myself now… Having this sense of security has really helped my overall well-being too. The knowledge that I had that help if needed made me seek less help, that’s for sure” (5)*

*1.2 Avoiding care-related suffering*

Direct access without involving the emergency department (ED) was a key benefit of the model. The ED was described as an unsupportive and overcrowded environment that intensified psychological distress. Patients often had to repeat their medical history to multiple healthcare staff, which many found exhausting. Prolonged waits for psychiatric assessment added further stress. Several participants felt unwelcome and reported feeling questioned or dismissed. Before access to self-admission, participants often avoided seeking help until emergency care was unavoidable. Being denied inpatient care at the ED often resulting in a prolonged recovery period. The rejection was described as hurtful and increased suicidality and self-harm was described.

*“If I feel like I want to, like everything is starting to fall apart, then I want to be sure that if I go to [the hospital] or somewhere else, the doctor there will see it the same way I do. Because it's really difficult to go in and then have them make a different assessment, and suddenly you're left standing there” (16)*

*1.3 Relief for relatives*

Some participants reported that their relatives felt reassured by their improved access to inpatient care. Moreover, the agreement appeared to ease their worry, and emotional burden of responsibility.

*“Yes, I notice it with my sons, they are 14 and 24 years old, and they are not as nervous, worried, or anxious anymore. It’s like they’ve been able to take a step back and avoid the strongest worry—knowing that I’m doing something, you know” (1)*

Simultaneously, it was reported that the program encouraged relatives to become more engaged in the patient’s mental healthcare, fostering a deeper understanding of the patient’s needs. However, two of the participants expressed insufficient support and understanding from relatives in connection with the generic self-admission model and suggested that healthcare should do more to involve relatives in the planning of the agreement and during self-admission.

*2. Care that supports*

According to the participants, access to self-admission provided greater opportunity for recovery by enabling early admission and individualized support during inpatient care. Self-admission was described as a more flexible form of care, with the adaptability being a key to early recovery and everyday functioning. These experiences are described in two subthemes.

*2.1 Bridging care and everyday life*

Being able to initiate inpatient care at an earlier stage, resulted in a shorter need for inpatient care and shorter recovery process afterwards. This had significant impact on the participants everyday lives. A common reason for using self-admission was the need to resume daily routines or overcome isolation at home. The possibility of choosing when to be admitted and flexible inpatient care was stressed as important. Some had previously avoided inpatient care due to fears of being confined to a ward with limited control over decisions such as permission and discharge. Instead, self-admission aligned with the participants’ needs and situation. For example, the importance of maintaining employment during periods of strained home situations. One participant highlighted the benefit of being able to adjust work around admission, occasionally even working during her inpatient stay. Prior to the generic self-admission model, leaving the ward required explicit permission from a psychiatrist.

*"Yeah, then they open the doors for me, and I tell them roughly when I'll be back. So I went off and worked, and then went straight to [hospital]. And this feels so safe, because sometimes when I’ve been working and then thought about [partner], I’ve felt like the anger flares up or 'No, I’m definitely not in balance.' Then it’s been really nice to just land at [hospital]” (14)*

Participants described the benefit of maintaining stabilizing factors such as work, regular activities and appointments as essential for recovery and well-being. The flexibility of the self-admission program also allowed parents to coordinate care for their children with the other caregiver or family members, ensuring a smoother transition and reducing stress for everyone involved.

*“…the day before, [name of youngest son] was supposed to go to him. But then I called my older son and asked if [the youngest son] could stay there. And he could. So that I was able to admit myself one day earlier than I had planned. […..] And then I sort of felt that, no, instead of doing something stupid, I’ll call and check that there is a bed. And that felt good, because I don’t really know if I would have called the outpatient care as early as I chose to admit myself.” (1)*

*2.2 Supportive inpatient care*

Some participants expressed relief at being able to manage symptoms of ill-health in an inpatient setting rather than at home, where they might feel exposed in front of family, friends, or neighbours. The generic self-admission model offered independence, discretion and anonymity during challenging times. One participant, who had a child at home, described how the self-admission program allowed her to avoid experiencing distressing symptoms in front of the child.

*“Last year was very chaotic... having a lot of anxiety, like I do, combined with the pressure of having a stepson every other week. [It] has at times been completely overwhelming, and there’s been a lot of arguing with my husband. Last fall was just a blanket of darkness, and when it got to be too much, it was such a relief to be able to leave the house, get help with basic things, like having meals served… if my stepson is home, it’s important that I don't act on impulses and self-harm, at least not in front of him.” (4)*

The interpersonal aspect was seen as central to the value of self-admission. Supportive conversations and welcoming staff fostered the sense of safety, respect, and recovery, while unsupportive encounters reduced the perceived benefits of self-admission.

*“I go there and... it’s a space where I stay in one place. There’s a lot of staff, and it’s mainly the presence of the staff that provides me with support in some way. I can talk, bounce some thoughts off them if I want, and get some company. That makes things calmer. I think it’s mainly the physical support that makes it calmer... The voices—the talking—stop. Maybe not completely, but almost. It calms down at least, and I get a little more peace.” (8)*

*3. Facilitating recovery*

Access to the generic self-admission model strengthened participants’ sense of self-determination. For several, the increased autonomy fostered personal growth, improved self-awareness and the ability to assess early warning signs, as well as the successful use of strategies that resulted in empowerment. This is presented in three sub-themes.

*3.1 Strengthening and challenging autonomy*

Most participants felt that access to self-admission enhanced their autonomy and involvement in care. Particularly the ability to initiate admission themselves significantly enhanced their sense of control and safety. This, in turn, fostered greater trust in themselves as well as in the mental health services. Access to self-admission was seen as a form of validation from mental health services - an acknowledgement of patients’ ability to take responsibility for their own health and care. This recognition contributed to feelings of dignity, self-confidence and self-worth.

*“It is positive, stabilizing. I would say that it is. I have a goal that…I really see self-admission as a big victory. It means that it doesn't risk becoming dangerous and that I don’t need to go through the emergency room. And it's also for my self-confidence, my self-trust. Because that is something that really gets totally destroyed, it's one's relationship with oneself when you get so sick, as I have been through the years. You start to not trust yourself at all. Your thoughts and feelings, you don't trust them at all.” (9)*

However, some participants also reported challenges related to increased autonomy and access to self-admission. Concerns were expressed regarding the availability of self-admission when needed. Moreover, some participants found the increased autonomy itself to be difficult, especially during the initial phase of the program, occasionally experiencing uncertainty about when—or whether—it was appropriate to initiate self-admission. Their concerns were often rooted in comparisons with other patients accompanied by a fear of misusing or occupying a hospital bed that someone else might need more urgently.

*“Another thing I can experience is that I can feel a bit like, "Oh! I'm in self-admission now, do I really have the right to do this? Am I taking someone else's place? Does this cost society? Am I really allowed to do this?". I can think, "What if someone thinks that?” (8)*

This occasionally led to participants being hesitant to use self-admission, even when they felt a need for inpatient care. One participant expressed doubt about admitting herself without a psychiatrist’s approval, leading to uncertainty about whether others saw her as undeserving of inpatient care. The participant further explained that, in the absence of external validation, it was difficult to trust her own judgement regarding the need for inpatient care. As a result, she had not yet found the program helpful, though she believed it might become beneficial once more familiar with the process.

*3.2 Promoting self-awareness and help-seeking*

Participant described a shift in the approach from health care staff, from doubting the severity of their struggles to feeling supported. Reassurance and encouragement from the staff to seek inpatient care earlier alleviated feelings of self-doubt and made it easier for them to reach out for professional support. The opportunity to access inpatient care at an earlier stage increased participants’ awareness of early signs of deterioration and when to seek support. Together with staff encouragement to seek help early, this supported participants in avoiding acute crises, experiencing a positive spiral with increased confidence and improved ability to express and act according to their needs. Participants described how access to self-admission supported them in daring to seek help and in communicating how they felt. One participant described more honest communication about suicidality because of participating in the self-admission program. This contributed to a greater sense of safety. However, some participants noted that changing their help-seeking behavior was challenging, particularly during the initial period after gaining access to self-admission and described this as part of an ongoing learning process.

*“... I have become better at calling perhaps earlier than when I self-harm, but in the beginning, it was mostly that I had already crossed the point where I had self-harmed, and then I realized, it’s a bit like I then realize “hospital,” but it has been good. I have a crisis plan that I am more or less good at following, and... I know that if I have strong impulses to self-harm, that’s when I should contact the department so that I don’t do it” (4)*

*3.3 Empowerment and confidence in own resources*

Participants described how the self-admission program encouraged them to take greater responsibility for their own well-being. The strategies outlined in the crisis plan and the agreement served as helpful reminders of actions to take before initiating self-admission.

*“And... there too, overall, the whole thing with the crisis plan... I remember when we first wrote it, I felt like, "What is this piece of paper going to do?" Like, yeah, I know what I should do, or can do. But it makes a big difference, at least for me. I experience it as... Well, you have to try the things, or you have to try to do the things that are written down because otherwise... yeah, you haven’t done your part, your part of the agreement or whatever you want to call it” (16)*

This process fostered increased self-awareness and more consistent use of early coping strategies, which in turn strengthened participants’ trust in themselves and their resources. One participant described how this helped her become more aware of, and actively strengthen, her protective factors such as maintaining regular eating and sleeping routines. Another strategy was to contact relatives more frequently and at an earlier stage, which often led to spending more time with family and friends. This, in turn, contributed to a greater sense of safety within their social network, making it easier to continue seeking their support. This strengthened the motivation and enhanced overall well-being.

*“I ask myself about admission much earlier, it also means that I turn to other things earlier. So, when I start feeling like it might be time to check in, I usually first reach out to friends or family, maybe visit them for a day and see if that helps… So, that’s probably what’s led to me not needing admission either. Um, so in a way, it has helped me build up my protective factors much more because I have it like this. Because it's not about having to make the decision when I’m at my lowest point.” (16)*

Several participants noted they had gained more control, improving their ability to handle challenges and persevere in difficult situations. Moreover, knowing that help was readily available encouraged several of the participants to challenge themselves more in daily life, such as going out more and exposing themselves to emotionally demanding situations.

*“It’s hard to know because it’s in the back of my mind. So maybe I go out a bit more, I can go out and sit at a café and feel that, if this doesn’t work and it gets too bad, I can go in. And most of the time, when I get the most anxiety, I just go home and sleep, put the pillow over my head. That’s usually the most effective. And at the same time, it’s nice to know that, yes… I might dare more, expose myself more to situations I might not actually want to” (2)*

## Discussion

Most participants reported that access to the generic self-admission model made them feel more secure and enhanced their awareness and capacity to cope with challenges more constructively. Access to self-admission supported patients in applying strategies outlined in their crisis plans, thereby helping them to prevent emotional breakdowns and decreasing the need for emergency care. The findings align with previous research on self-admission models, though certain aspects are more strongly emphasized in the present study and will be discussed further below.

Notably, the centrality of relatives’ involvement and the importance of these relationships emerged clearly, albeit expressed in different ways. The generic self-admission model was seen as supportive in managing everyday challenges and in promoting health-processes and well-being by reinforcing both internal and external resources. Knowing that self-admission was available as a backup was associated with increased confidence among participants and their relatives, and, for some, improved relationships with close relatives. In some cases, relatives reached out more frequently to offer support. The improvements also appeared to be associated with patients initiating more contact with relatives, either to prevent mental health deterioration or due to increased feelings of security that enabled engagement in previously avoided situations. Similar findings have been reported in studies focusing on relatives’ perspective of self-admission models (Hultsjo, Appelfeldt et al., 2023; Hultsjo, Rosenlund et al., 2023; Lindkvist et al., 2024). While relatives of patients receiving standard psychiatric care frequently report concern, guilt, and a sense of responsibility for the patient—factors that may negatively affect their well-being - access to supportive care for the patient has been found to enhance relatives’ capacity to respond to the patient’s deteriorating condition and initiate referrals to appropriate services (Corchon et al., 2022; Johansson et al., 2014). Similarly, access to self-admission seems to facilitate relatives’ ability and confidence to support the patient, potentially strengthening their relationships and improving support for both the patient and the relative.

However, a few participants in the current study reported a lack of support and understanding from relatives. Despite relatives involvement being a core element of person-centred care (Khosravi et al., 2024) studies of standard psychiatric care report that relatives often feel excluded, poorly informed and insufficiently supported by healthcare services (Aass et al., 2022; Staniszewska et al., 2019; Sugiura et al., 2020). Relative involvement can be crucial for enabling early interventions and preventing compulsory care, provided they receive clear information about available support and treatment. Well-informed relatives can more readily identify early deterioration and help maintain the patient’s trust in healthcare (Sugiura et al., 2020). While the generic self-admission model may promote greater involvement of relatives, healthcare providers are encouraged to implement structured routines to support this, including involving relatives—with the patient’s consent—in the planning and follow-up of the agreement.

Participants reported increased autonomy associated with access to the generic self-admission model, aligning with findings from previous research on self-admission models (Värnå et al., 2025). In the current study, increased autonomy emerged as both beneficial and challenging for participants. The opportunity to access inpatient care based on patients’ own decisions enabled a more effective and individualized use of healthcare services, better aligned with the patients’ needs and preferences. For instance, self-admission enabled patients to take a beneficial inpatient break without disrupting important commitments. This study highlighted that increased trust in, and from, staff and the healthcare system strengthened participants’ autonomy and self-efficacy. Improved self-assessment and use of crisis-plan strategies after access to self-admission further enhanced their sense of control and reinforced confidence in their coping abilities and social support network, thereby facilitating recovery. In a systematic review of 97 studies, five core concepts central to personal recovery in mental health were identified. These, described as the CHIME framework, can all be related to our findings. Self-admission reinforced *Connectedness* by reinforcing therapeutic relationships and timely access to care. The participants also described how the encouragement and recognition of progress—both self-perceived and acknowledged by others—fostered *Hope* and a positive *Identity.* Furthermore, by enabling individuals to make active care related decisions aligned with personal values and meaningful activities, it contributes to *Meaning* and *Empowerment* (Leamy et al., 2011). The supportive structure of the generic self-admission model, as a person-driven process grounded in belonging and self-agency, may also promote social inclusion—a growth process fostered by increased opportunities for interaction and participation, with respect for individual expectations and autonomy (Cobigo et al., 2012).

However, this focus on inclusion based on personal choices and needs rather than assimilation to dominant norms is complex (Cobigo et al., 2012). For example, some participants found it challenging to manage enhanced responsibility and autonomy when they were expected to assess their own need for care without confirmation from healthcare staff. This may reflect familiarity with traditional, medically oriented care models, shaped by participants’ previous experiences. Alternatively, the generic self-admission model may not have been well suited to these individuals’ needs for reasons not captured in this study. Some participants’ experiences may be understood in relation to stigma, which may occur when individuals with mental health problems internalize negative stereotypes and prejudices held by the general public (Ihalainen et al., 2022). According to Bergamin and colleagues (Bergamin et al., 2022), stigma can hinder autonomy in mental health care by limiting individuals’ ability to make self-directed choices aligned with their values. It may also prevent them from recognizing their mental health needs, seeking care, and engaging in appropriate treatment, thereby worsening their condition and further undermining their autonomy (Bergamin et al., 2022). Our results concern being seen and judged by others during mental health crises, for example in the ED where unhelpful encounters with healthcare staff may reinforce stigma. Shifting from reactive to proactive inpatient care may help counter such stigma by promoting earlier, more normalized help-seeking. However, the transition to independently deciding on the need for inpatient care can be challenging. It is therefore important that healthcare providers are aware of challenges related to stigma and offer appropriate support to self-admission patients, particularly during the initial period after they gain access to the model.

This study adds to existing literature by exploring a generic self-admission model from the patient perspective. To our knowledge, this type of model has not previously been implemented or evaluated. It is therefore important to assess whether it yields outcomes comparable to previous studies on self-admission models—an indication supported by our findings. Based on this study, a generic self-admission model appears to offer support for patients with diverse psychiatric conditions and challenges. The model is used, for instance, for crisis management and self-harm prevention, to regain and maintain daily structure, and to cope with different psychiatric symptoms such as anxiety and auditory hallucinations. Unlike diagnosis-specific models, the generic self-admission model is based on general criteria, allowing access to be tailored to each patient’s individual needs and circumstances. This aligns more closely with person-centred care (McCance & McCormack, 2025).

Several key implementation outcomes are relevant when evaluating interventions (Proctor et al., 2011). This study focused on patients' experiences of an implemented intervention, an important perspective for understanding its practical impact. The generic self-admission model was described as meaningful, supportive, and responsive to individual needs, indicating high acceptability. Participants emphasized the model’s compatibility with individual needs, reflecting high appropriateness. The model’s flexible, low-threshold, and autonomy-promoting design aligns well with the goals of mental healthcare, as well as with recovery-oriented and person-centred principles, thereby supporting its relevance and potential for sustainable integration (Proctor et al., 2011). However, further research is needed to evaluate the implementation from healthcare staff’s perspective, as well as to explore the mechanisms of action underlying the generic self-admission model and its impact on healthcare needs, utilization, and costs.

### Methodological considerations

Several aspects of the study design contribute to its strengths. The inclusion of participants with diverse backgrounds in terms of gender, age, diagnosis, and prior experiences with self-admission hospitalization enabled a broad range of perspectives on the model. In addition, several aspects contributed to the study’s overall trustworthiness. During the analytical process, investigator triangulation was performed by two authors with differing professional backgrounds and experience with self-admission models, who independently conducted the initial coding. The comparison of codes revealed a high level of consistency, strengthening the study’s credibility. These discussions supported reflexivity and further strengthened the analytical depth and credibility. The consistency of our findings with previous studies supports both credibility and confirmability of the analysis (Korstjens & Moser, 2018). However, although several core components are shared across national and international self-admission models, variations between models exist and may shape participants’ experiences of access to self-admission and thereby study results.

Among the co-authors and interviewers, there was considerable variation in experience related to working with self-admission models. This diversity presents both strengths and challenges in the interview context. Prior clinical experience with the model can deepen the interviewer’s understanding of participants’ situations and support more responsive interviewing, yet it may also introduce preconceptions that could bias data interpretation. Potential bias was mitigated through regular research team discussions on preliminary findings, interpretations, expectations, and prior experiences, which enhanced analytical depth and credibility. These dialogues supported reflexivity and contributed to the integrity and trustworthiness of the research. The audit trail was maintained throughout the research process, supporting the study’s dependability and confirmability.

One limitation of the study is that the involvement of four interviewers may have introduced variability in questioning and prompting, thereby influencing the findings. To minimize this risk, the research team engaged in regular discussions to reflect on their experiences, address emotional responses, provide mutual feedback, and promote consistency in interviewing practices, which is important when multiple interviewers are involved (Demirci, 2024). The systematic use of reflexive journaling and iterative process, moving repeatedly between raw data, analytic phases, and emerging findings, strengthened the critical examination, transparency and confirmability.

Half of the interviews were conducted by telephone. Although face-to-face interviews may facilitate engagement and tend to last longer, telephone interviews can yield data of comparable depth and promote openness on sensitive topics, potentially enhancing credibility (Irvine et al., 2012; Novick, 2008). Based on this, and to ensure accessibility and inclusion, participants were allowed to choose the interview format.

The analysis focused on how patients perceive their access to self-admission and how this access influenced their everyday lives, regardless of whether they had ever used the intervention to admit themselves to inpatient care. We considered these differing experiences and recognized that patients’ perceptions may have been shaped by whether they had practical experience with the intervention. Despite varied backgrounds, several common themes were identified. This aligns with prior research showing that availability of self-admission can be supportive even when patients do not actually use it (Värnå et al., 2025). The authors reflective and restrained stance throughout the analytic process ensured a careful and respectful interpretation of participants’ narratives, particularly given their vulnerability and the importance of allowing all voices to be heard (Pertega et al., 2025).

Because recruitment partly relied on staff engagement, patients who were involved in their care—or perceived as more suitable—may have been more likely to be informed about the study, potentially narrowing the range of experiences represented and thereby affecting the study’s credibility. Transparency in the recruitment process and reflexive discussions within the research team were employed to mitigate and critically reflect on potential bias.

Data collection was conducted over a period of 16 months. This reflected the gradual implementation of self-admission across participating services, practical considerations related to recruitment within standard clinical care, and periods of limited researcher availability. While the extended data collection period may have captured experiences at different stages of implementation, this was considered appropriate for exploring patients’ experiences of access to self-admission in a real-world clinical context.

## Conclusion

This study adds to the growing evidence that self-admission models offer meaningful support for patients with severe mental health conditions. Participants found the generic self-admission model highly valued, describing a sense of security that reduced suffering and supported recovery by strengthening self-agency. The model contributed to improved coping strategies, reduced need for inpatient care, and better relationships where participants noted reduced strain on relatives. The agreement and crisis plan were perceived as supportive and being able to maintain stabilizing factors such as work and everyday routines by using self-admission was described as essential for recovery. Some participants reported challenges related to increased responsibility, and some emphasized the need for greater involvement of relatives - whose experiences with the generic self-admission model remain unexplored. Further research is needed to examine the model’s broader effects on healthcare needs, utilization, and costs.

The overall positive findings indicate that the generic self-admission model is an acceptable and helpful intervention that supports autonomy and recovery. Its transdiagnostic and flexible structure may promote broader, equitable access to crisis support and mental health care aligned with patients’ rights, regardless of diagnosis. The generic self-admission model may therefore contribute to development of mental healthcare practices that more effectively address patients’ needs and life circumstances during various mental health conditions, and to a more person-centred care.

## Supplementary Material

Appendix_1.docx Appendix_1.docx

## Data Availability

The participants of this study did not give written consent for their data to be shared publicly, so due to the sensitive nature of the research supporting data is not available.

## References

[cit0001] Aass, L. K., Moen, O. L., Skundberg-Kletthagen, H., Lundqvist, L. O., & Schroder, A. (2022). Family support and quality of community mental health care: Perspectives from families living with mental illness. *Journal of Clinical Nursing*, *31*(7-8), 935–948. 10.1111/jocn.15948 34240499

[cit0002] Ahlstrand, A., Mishina, K., Elomaa-Krapu, M., & Joronen, K. (2024). Consumer involvement and guiding frameworks in mental healthcare: An integrative literature review. *International Journal of Mental Health Nursing*, *33*(5), 1227–1241. 10.1111/inm.13343 38706160

[cit0003] Bergamin, J., Luigjes, J., Kiverstein, J., Bockting, C. L., & Denys, D. (2022). Defining autonomy in psychiatry. *Frontiers in Psychiatry*, *13*. 801415. 10.3389/fpsyt.2022.801415 35711601 PMC9197585

[cit0004] Braun, V., & Clarke, V. (2006). Using thematic analysis in psychology. *Qualitative Research in Psychology*, *3*(2), 77–101. 10.1191/1478088706qp063oa

[cit0005] Braun, V., & Clarke, V. (2013). Successful qualitative reserach - a practical guide for beginners (1st ed.). SAGE.

[cit0006] Braun, V., & Clarke, V. (2019). To saturate or not to saturate? Questioning data saturation as a useful concept for thematic analysis and sample-size rationales. *Qualitative Research in Sport, Exercise and Health*, *13*(2), 201–216. 10.1080/2159676x.2019.1704846

[cit0007] Cobigo, V., Ouellette-Kuntz, H., Lysaght, R., & Martin, L. (2012). Shifting our conceptualization of social inclusion. *Stigma Research and Action*, *2*(2), 75–84. 10.5463/sra.v1i3.45

[cit0008] Corchon, S., Sanchez-Martinez, V., & Cauli, O. (2022). Perceived mental health and emotional trajectories of long-term family caregivers of persons with mental conditions: A mixed-methods study. *Archives of Psychiatric Nursing*, *41*, 105–113. 10.1016/j.apnu.2022.07.015 36428037

[cit0009] Daukantaite, D., Lindkvist, R. M., Lantto, R., & Westling, S. (2025). Brief admission by self-referral: A 4-Year follow-up on utilisation patterns and experiences. *International Journal of Mental Health Nursing*, *34*(4), e70091. 10.1111/inm.70091 40635140 PMC12241490

[cit0010] Demirci, J. R. (2024). About research: Conducting better qualitative interviews. *Journal of Human Lactation : Official Journal of International Lactation Consultant Association*, *40*(1), 21–24. 10.1177/08903344231213651 37994717

[cit0011] Eckerström, J., Flyckt, L., Carlborg, A., Jayaram-Lindström, N., & Perseius, K.-I. (2020). Brief admission for patients with emotional instability and self-harm: A qualitative analysis of patients’ experiences during crisis. *International Journal of Mental Health Nursing*, *29*, 962–971. 10.1111/inm.12736 32406168

[cit0012] Eckerström, J., Allenius, E., Helleman, M., Flyckt, L., Perseius, K. I., & Omerov, P. (2019). Brief admission (BA) for patients with emotional instability and self-harm: nurses' perspectives - person-centred care in clinical practice. *International Journal of Qualitative Studies on Health and Well-Being*, *14*(1), 1667133. 10.1080/17482631.2019.1667133 31526310 PMC6758609

[cit0013] Eckerström, J., Rosendahl, I., Lindkvist, R.-M., Amin, R., Carlborg, A., Flyckt, L., & Jayaram-Lindström, N. (2024). Effects of Patient- initiated brief admissions on psychiatric care consumption in borderline personality disorder: A register-based study. *International Journal of Mental Health Nursing*, *33*, 2080–2089. 10.1111/inm.13371 38855833

[cit0014] Forsgren, E., Feldthusen, C., Wallstrom, S., Bjorkman, I., Bergholtz, J., Friberg, F., & Ohlen, J. (2025). Learning from the implementation of person-centred care: A meta-synthesis of research related to the gothenburg framework. *Front Health Serv*, *5*, 1589502. 10.3389/frhs.2025.1589502 40666166 PMC12259557

[cit0015] Gabrielsson, S., Tuvesson, H., Wiklund Gustin, L., & Jormfeldt, H. (2020). Positioning psychiatric and mental health nursing as a transformative force in health care. *Issues in Mental Health Nursing*, *41*(11), 976–984. 10.1080/01612840.2020.1756009 32584618

[cit0016] Hagsäter, M., Ohlsson, M., Casanovas Roca, M., Sjöstedt, A., & Hieronymus, F. (2025). Patient-initiated brief admission: A single site eight-year retrospective cohort study. *Acta Neuropsychiatr*, *37*, e80. 10.1017/neu.2025.10031 40855778 PMC13130303

[cit0017] Hälso- och sjukvårdslag [HSL] (SFS 2017:30). https://www.riksdagen.se/sv/dokument-lagar/dokument/svensk-forfattningssamling/halso--och-sjukvardslag-201730_sfs-2017-30

[cit0018] Harris, B., Beurmann, R., Fagien, S., & Shattell, M. M. (2016). Patients' experiences of psychiatric care in emergency departments: A secondary analysis. *International Emergency Nursing*, *26*, 14–19. 10.1016/j.ienj.2015.09.004 26459607

[cit0019] Helleman, M., Goossens, P. J., Kaasenbrood, A., & van Achterberg, T. (2014a). Evidence base and components of brief admission as an intervention for patients with borderline personality disorder: A review of the literature. *Perspectives in Psychiatric Care*, *50*(1), 65–75. 10.1111/ppc.12023 24387616

[cit0020] Hormazabal-Salgado, R., Whitehead, D., Osman, A. D., & Hills, D. (2024). Person-centred decision-making in mental health: A scoping review. *Issues in Mental Health Nursing*, *45*(3), 294–310. 10.1080/01612840.2023.2288181 38232185

[cit0021] Hultsjo, S., Appelfeldt, A., Wardig, R., & Cederqvist, J. (2023). Don t set us aside!Experiences of families of people with BPD who have access to brief admission:A phenomenological perspective. *International Journal of Qualitative Studies on Health and Well-Being*, *18*(1. 2152943. 10.1080/17482631.2022.2152943 36476045 PMC9733683

[cit0022] Hultsjo, S., Rosenlund, H., Wadsten, L., & Wardig, R. (2023). Relatives' experiences of brief admission in borderline personality disorder and self-harming behaviour. *Nursing Open*, *10*(4), 2338–2348. 10.1002/nop2.1487 36403239 PMC10006650

[cit0023] Ihalainen, N., Loyttyniemi, E., & Valimaki, M. (2022). Self-stigma among clients of outpatient psychiatric clinics: A cross-sectional survey. *PLoS One*, *17*(7), e0269465. 10.1371/journal.pone.0269465 35776719 PMC9249178

[cit0024] Irvine, A., Drew, P., & Sainsbury, R. (2012). Am I not answering your questions properly?’ clarification, adequacy and responsiveness in semi-structured telephone and face-to-face interviews. *Qualitative Research*, *13*(1), 87–106. 10.1177/1468794112439086). ‘

[cit0025] Johansson, A., Andershed, B., & Anderzen-Carlsson, A. (2014). Conceptions of mental health care--from the perspective of parents' of adult children suffering from mental illness. *Scandinavian Journal of Caring Sciences*, *28*(3), 496–504. 10.1111/scs.12074 23980612

[cit0026] Khosravi, M., Azar, G., & Izadi, R. (2024). Principles and elements of patient-centredness in mental health services: A thematic analysis of a systematic review of reviews. *BMJ Open Quality*, *13*(3), e002719. 10.1136/bmjoq-2023-002719 PMC11227821 38960446

[cit0027] Korstjens, I., & Moser, A. (2018). Series: Practical guidance to qualitative research. Part 4: Trustworthiness and publishing. *The European Journal of General Practice*, *24*(1), 120–124. 10.1080/13814788.2017.1375092 29202616 PMC8816392

[cit0028] Leamy, M., Bird, V., Boutillier, C., Williams, J., & Slade, M. (2011). Conceptual framework for personal recovery in mental health: Systematic review and narrative synthesis. *British Journal of Psychiatry*, *199*(6), 445–452. 10.1192/bjp.bp.110.083733 22130746

[cit0029] Lindkvist, R. M., Eckerström, J., Landgren, K., & Westling, S. (2024). Brief admission by self-referral for individuals with self-harm and suicidal ideation: A qualitative study based on focus groups exploring relatives' experiences. *International Journal of Qualitative Studies on Health and Well-Being*, *19*(1), 2353460. 10.1080/17482631.2024.2353460 38739443 PMC11095277

[cit0030] McCance, T., & McCormack, B. (2025). The person-centred nursing framework: A mid-range theory for nursing practice. *J Res Nurs*, *30*(1), 47–60. 10.1177/17449871241281428 PMC11907491 40093819

[cit0031] Molin, J., Graneheim, U. H., & Lindgren, B. M. (2016). Quality of interactions influences everyday life in psychiatric inpatient care--patients' perspectives. *International Journal of Qualitative Studies on Health and Well-Being*, *11*, 29897. 10.3402/qhw.v11.29897 26806313 PMC4724788

[cit0032] Mortimer-Jones, S., Morrison, P., Munib, A., Paolucci, F., Neale, S., Bostwick, A., & Hungerford, C. (2016). Recovery and borderline personality disorder: A description of the innovative open borders program. *Issues in Mental Health Nursing*, *37*(9), 624–630. 10.1080/01612840.2016.1191565 27327362

[cit0033] Mortimer-Jones, S., Morrison, P., Munib, A., Paolucci, F., Neale, S., Hellewell, A., Sinwan, J., & Hungerford, C. (2019). Staff and client perspectives of the open borders programme for people with borderline personality disorder. *International Journal of Mental Health Nursing*, *28*, 971–979. 10.1111/inm.12602 31081282

[cit0034] Novick, G. (2008). Is there a bias against telephone interviews in qualitative research? *Research in Nursing & Health*, *31*(4), 391–398. 10.1002/nur.20259 18203128 PMC3238794

[cit0035] NSPH. (2026). The Swedish Partnership for Mental Health, NSPH. Retrieved 2026 February 17 from https://NSPH.se/om-oss/in-english/

[cit0036] Nyttingnes, O., Benth, J. S., & Ruud, T. (2021). Patient-controlled admission contracts: A longitudinal study of patient evaluations. *BMC Health Services Research*, *21*(36). 10.1186/s12913-020-06033-4 PMC7791868 33413337

[cit0037] Paaske, L. S., Sopina, L., Olsen, K. R., Thomsen, C. T., Benros, M. E., Nordentoft, M., & Hastrup, L. H. (2021). The impact of patient-controlled hospital admissions among patients with severe mental disorders on health care cost: A nationwide register-based cohort study using quasi-experimental design. *Journal of Psychiatric Research*, *144*, 331–337. 10.1016/j.jpsychires.2021.10.032 34737122

[cit0038] Pertega, E., Holmberg, C., Dahlberg, K., & Dahlberg, H. (2025). Lifeworld-led research: A phenomenological approach to grant experts by experience in vulnerable positions their right to participate in healthcare research. *International Journal of Qualitative Studies on Health and Well-Being*, *20*(1), 2522875. 10.1080/17482631.2025.2522875 40571718 PMC12203683

[cit0039] Price, B. (2002). Laddered questions and qualitative data research interviews. *Journal of Advanced Nursing (Oxford)*, *37*(3), 273–281. 10.1046/j.1365-2648.2002.02086.x 11851798

[cit0040] Proctor, E., Silmere, H., Raghavan, R., Hovmand, P., Aarons, G., Bunger, A., Griffey, R., & Hensley, M. (2011). Outcomes for implementation research: Conceptual distinctions, measurement challenges, and research agenda. *Administration and Policy in Mental Health*, *38*(2), 65–76. 10.1007/s10488-010-0319-7 20957426 PMC3068522

[cit0041] Region Stockholm. (2025, February 24). Så ökade befolkningen 2024. https://www.regionstockholm.se/nyheter/2025/02/sa-okade-befolkningen-2024/

[cit0042] Sigrunarson, V., Moljord, I. E., Steinsbekk, A., Eriksen, L., & Morken, G. (2017). A randomized controlled trial comparing self-referral to inpatient treatment and treatment as usual in patients with severe mental disorders. *Nordic Journal of Psychiatry*, *71*(2), 120–125. 10.1080/08039488.2016.1240231 27739334

[cit0043] Silva, B., Bachelard, M., Rosselet Amoussou, J., Martinez, D., Bonalumi, C., Bonsack, C., Golay, P., & Morandi, S. (2023). Feeling coerced during voluntary and involuntary psychiatric hospitalisation: A review and meta-aggregation of qualitative studies. *Heliyon*, *9*(2), e13420. 10.1016/j.heliyon.2023.e13420 36820044 PMC9937983

[cit0044] Skott, M., Durbeej, N., Smitmanis-Lyle, M., Hellner, C., Allenius, E., Salomonsson, S., Lundgren, T., Jayaram-Lindström, N., & Rozental, A. (2021). Patient-controlled admissions to inpatient care: A twelve-month naturalistic study of patients with schizophrenia spectrum diagnoses and the effects on admissions to and days in inpatient care. *BMC Health Services Research*, *21*, 598. 10.1186/s12913-021-06617-8 34162390 PMC8223388

[cit0045] Smitmanis Lyle, M., Allenius, E., Salomonsson, S., Björkdahl, A., Strand, M., Flyckt, L., Hellner, C., Lundgren, T., Jayaram-Lindstrom, N., & Rozental, A. (2022). What are the effects of implementing patient-controlled admissions in inpatient care? A study protocol of a large-scale implementation and naturalistic evaluation for adult and adolescent patients with severe psychiatric conditions throughout region Stockholm. *BMJ Open*, *12*(8), e065770. 10.1136/bmjopen-2022-065770 PMC9386218 35973700

[cit0046] Socialstyrelsen. (2021). Utvärdering av metoden självvald inläggning - Kartläggning och analys av metoden inom svensk psykiatri (2021-11-7662). https://www.socialstyrelsen.se/globalassets/sharepoint-dokument/artikelkatalog/ovrigt/2021-11-7662.pdf

[cit0047] Socialstyrelsen. (2025, April 3). Personcentrering vid psykisk funktionsnedsättning och stora behov. https://www.socialstyrelsen.se/kunskapsstod-och-regler/omraden/psykisk-ohalsa/personcentrering-vid-psykisk-funktionsnedsattning-och-stora-behov/

[cit0048] Socialstyrelsen. (2026, January 15). Heldygnsvård, tvångsvård och självvald inläggning. https://www.socialstyrelsen.se/kunskapsstod-och-regler/omraden/psykisk-ohalsa/heldygnsvard-och-tvangsvard/

[cit0049] Staniszewska, S., Mockford, C., Chadburn, G., Fenton, S. J., Bhui, K., Larkin, M., Newton, E., Crepaz-Keay, D., Griffiths, F., & Weich, S. (2019). Experiences of in-patient mental health services: Systematic review. *British Journal of Psychiatry*, *214*(6), 329–338. 10.1192/bjp.2019.22 30894243

[cit0050] Strand, M., Bulik, C. M., Gustafsson, S. A., von Hausswolff-Juhlin, Y., & Welch, E. (2020). Self-admission to inpatient treatment in anorexia nervosa: Impact on healthcare utilization, eating disorder morbidity, and quality of life. *The International Journal of Eating Disorders*, *53*(10), 1685–1695. 10.1002/eat.23346 32666605

[cit0051] Sugiura, K., Pertega, E., & Holmberg, C. (2020). Experiences of involuntary psychiatric admission decision-making: A systematic review and meta-synthesis of the perspectives of service users, informal carers, and professionals. *International Journal of Law and Psychiatry (Elmsford, NY)*, *73*, 101645. 10.1016/j.ijlp.2020.101645 33246221

[cit0052] Sveriges kommuner och regioner [SKR]. (2023). Vården i siffror. https://vardenisiffror.se/indikator/2e6faaf9-a97f-4ce8-b6a6-4afa29adce71?datefrom=2018-01-01&dateto=2023-12-31&gender&periodtype=year&relatedmeasuresbyentry=kallsystem&relatedmeasuresbyid=7a74e5d8-ce95-44c3-ad7b-5302a7fc8743&showtarget=false&units=se

[cit0053] Thomsen, C. T., Benros, M. E., Maltesen, T., Hastrup, L. H., Andersen, P. K., Giacco, D., & Nordentoft, M. (2018). Patient-controlled hospital admission for patients with severe mental disorders: A nationwide prospective multicentre study. *Acta Psychiatrica Scandinavica*, *137*(4), 355–363. 10.1111/acps.12868 29504127

[cit0054] Tong, A., Sainsbury, P., & Craig, J. (2007). Consolidated criteria for reporting qualitative research (COREQ): A 32-item checklist for interviews and focus groups. *International Journal for Quality in Health Care*, *19*(6), 349–357. 10.1093/intqhc/mzm042 17872937

[cit0055] Värnå, E., Nederman, J., Saliba-Gustafsson, E. A., & Eckerström, J. (2025). Patient experiences of patient-initiated brief admission in psychiatric care: A systematic review. *International Journal of Mental Health Nursing*, *34*(1), e13457. 10.1111/inm.13457 39462992 PMC11771678

[cit0056] Vogt, K. S., Baker, J., Kendal, S., Griffin, B. L., Mizen, E., Sharp, H., & Johnson, J. (2024). Safer, not Safe': Service Users' experiences of psychological safety in inpatient mental health wards in the United Kingdom. *International Journal of Mental Health Nursing*, *33*(6), 2227–2238. 10.1111/inm.13381). ' 39030900

[cit0057] Westling, S., Daukantaite, D., Liljedahl, S. I., Oh, Y., Westrin, A., Flyckt, L., & Helleman, M. (2019). Effect of brief admission to hospital by self-referral for individuals who self-harm and are at risk of suicide: A randomized clinical trial. *JAMA Netw Open*, *2*(6), e195463. 10.1001/jamanetworkopen.2019.5463 31173128 PMC6563573

