# Supplementary material for: From safety to agency: experiences of self-admission among patients with diverse mental health needs
Source: Int J Qual Stud Health Well-being. 2026 Mar 11;21(1):2641161. doi: 10.1080/17482631.2026.2641161 (PMC12981251; doi:10.1080/17482631.2026.2641161)
Supplement: Appendix_1.docx [file ZQHW_A_2641161_SM7107.docx]

**Appendix 1**

***Interview guide***

GENERALLY

I would like to hear more about you and your life situation. Please tell.

Is there anything you enjoy doing (interests)?

*What does a typical day look like for you?*

1. Is there anything that can stop you from doing those things?
2. How do you usually handle it? (What do you usually do then?)

SELF-ADMISSION

You have access to self-admission: Has it been helpful?

Has self-admission affected your everyday life (life situation)? In what way?

*Compared to before you had access to SI, there is some difference in:*

- *How do you manage your daily activities at home?*
- *How do you manage your daily activities work, study, daily activities?*
- *Has your relationship with your loved ones been affected?*
- *Your psychiatric difficulties have been affected?*

Can you give an example of how self-admission has affected your contact with psychiatric care?

*Compared to before you had access to SI, there is some difference in:*

- *Your relationship with psychiatric care?*
- *How do staff from psychiatric care (inpatient care/outpatient care) treat you/talk to you?*
- *How do you experience your role in the contact with healthcare?*

Have you admitted yourself for a self-admission?

*If yes:*

- *How have you experienced the opportunity for* self-admission *in those situations?*
- *What was the reason you chose to admit yourself?*
- *Whose idea was it for you to be admitted through* self-admission*?*
- *Have there been any obstacles when you wanted to admit yourself? If so, what were they?*
- *How has it worked for you to assess your own need for inpatient care?*
- *Have there been other occasions when you considered admission but chose not to? Why?*
- *In what way has admission been a support for you?*
- *In what way could the admission occasions be a better support for you?*

*If no:*

- *How come you haven’t used* self-admission*?*
- *Are there situations where you think* self-admission *could have been helpful, but you chose not to use it? If yes, why did you decide not to be admitted?*

What do you appreciate most about the content of the self-admission model?

In what way could self-admission be changed to better support you?

Have you ever been admitted under the Compulsory Psychiatric Act?

If yes, what is you experience of being cared for under this act?

Have you previously received standard voluntary psychiatric care?

If yes, how would you describe the difference between standard voluntary care and involuntary care?

How would you describe the differences between self-admission and your previous care experiences?
